# Identification of critical functional residues of receptor-like kinase ERECTA

**DOI:** 10.1093/jxb/erx022

**Published:** 2017-02-15

**Authors:** Pawel Z. Kosentka, Liang Zhang, Yonas A. Simon, Binita Satpathy, Richard Maradiaga, Omar Mitoubsi, Elena D. Shpak

**Affiliations:** 1 Department of Biochemistry, Cellular and Molecular Biology, University of Tennessee, Knoxville, TN 37996, USA

**Keywords:** Activation segment, ERECTA, kinase domain, LRR-RLK, phosphorylation, receptor kinase, signal transduction, site-directed mutagenesis, structure–functional analysis

## Abstract

In plants, extracellular signals are primarily sensed by plasma membrane-localized receptor-like kinases (RLKs). ERECTA is a leucine-rich repeat RLK that together with its paralogs ERECTA-like 1 (ERL1) and ERL2 regulates multiple aspects of plant development. ERECTA forms complexes with a range of co-receptors and senses secreted cysteine-rich small proteins from the EPF/EPFL family. Currently the mechanism of the cytoplasmic domain activation and transmission of the signal by ERECTA is unclear. To gain a better understanding we performed a structure–function analysis by introducing altered ERECTA genes into *erecta* and *erecta erl1 erl2* mutants. These experiments indicated that ERECTA’s ability to phosphorylate is functionally significant, and that while the cytoplasmic juxtamembrane domain is important for ERECTA function, the C-terminal tail is not. An analysis of multiple putative phosphorylation sites identified four amino acids in the activation segment of the kinase domain as functionally important. Homology of those residues to functionally significant amino acids in multiple other plant RLKs emphasizes similarities in RLK function. Specifically, our data predicts Thr812 as a primary site of phosphor-activation and potential inhibitory phosphorylation of Tyr815 and Tyr820. In addition, our experiments suggest that there are differences in the molecular mechanism of ERECTA function during regulation of stomata development and in elongation of above-ground organs.

## Highlight

A study of ERECTA cytoplasmic domain predicts a primary phosphorylation site in its activation segment and indicates that signal transduction by ERECTA differs in specific developmental processes.

## Introduction

Intercellular communications are essential for development of multicellular organisms where cell proliferation and differentiation must be cooperative and structured to attain a desired shape and function. Plants especially rely on intercellular communications as cell behavior is often position-dependent. To detect extracellular signals, plant cells have a large group of receptor-like kinases (RLKs). These receptors possess a structurally diverse extracellular ligand-sensing domain, a single-pass transmembrane domain, and a cytoplasmic serine/threonine/tyrosine kinase domain.

The ERECTA family (ERf) RLKs appeared early during land plant evolution and are involved in the regulation of multiple developmental processes (Villagarcia *et al.*, 2012, Shpak, 2013). During embryogenesis they stimulate cotyledon elongation (Chen and Shpak, 2014). Post-embryonically, ERfs promote growth of all above-ground organs (Shpak *et al.*, 2004). ERfs have been demonstrated to regulate stomata formation, the function of the shoot apical meristem (SAM), and the development of flowers (Shpak, 2013). In angiosperms, ERf consists of two or more genes, with Arabidopsis having three: *ERECTA* (*ER*), *ERECTA-like 1* (*ERL1*) and *ERECTA-like 2* (*ERL2*) (Shpak *et al.*, 2004, Villagarcia *et al.*, 2012). Although all three genes regulate above-ground organ elongation, they exhibit unequal redundancy. While *erecta* mutants have compact inflorescences due to shorter internodes and pedicels, single mutations in *erl1* and *erl2* confer no detectable phenotype (Torii *et al.*, 1996; Shpak *et al.*, 2004). Loss of all three genes leads to severe dwarfism (Shpak *et al.*, 2004). The reduced growth of above-ground plant organs in ERf mutants is associated with a decrease in the cell proliferation rate (Shpak *et al.*, 2003, 2004). An analysis of pedicel growth suggested that ERECTA accelerates the elongation of cells along the proximo-distal axis and shortens the duration of the cell cycle (Bundy *et al.*, 2012). The asymmetric redundancy of ERf receptors is also evident during stomata development. In the initial stage of the stomata development process, ERfs synergistically inhibit differentiation of protodermal cells into meristemoid mother cells. ERECTA plays a major role during this process, as an increased number of asymmetric cell divisions has been observed only in the *erecta* single mutant (Shpak *et al.*, 2005). Once meristemoids are formed, ERL1 and ERL2 inhibit their differentiation into guard mother cells (Shpak *et al.*, 2005). In the SAM, ERfs seem to be equally redundant; the receptors synergistically inhibit meristem enlargement, promote leaf initiation, and contribute to establishment of phyllotaxy (Chen *et al.*, 2013; Uchida *et al.*, 2013). Finally, ERfs play an important role in the regulation of ovule and early anther development (Pillitteri *et al.*, 2007; Hord *et al.*, 2008; Bemis *et al.*, 2013). The *er erl1 erl2* mutant is sterile with compromised male and female fertility (Shpak *et al.*, 2004).

ERECTA, ERL1, and ERL2 receptors form homo- and heterodimers (Lee *et al.*, 2012). They also make complexes with SOMATIC EMBRYOGENESIS RECEPTOR KINASEs (SERKs) and with the transmembrane receptor-like protein TOO MANY MOUTHS (TMM) (Lee *et al.*, 2012; Meng *et al.*, 2015). The activity of ERf receptors is regulated by a family of secreted cysteine-rich small proteins from the EPF/EPFL family, which can function as agonists or antagonists (Shimada *et al.*, 2011). A MAP kinase cascade consisting of YODA, MKK4, MKK5, MPK3, and MPK6 functions downstream (Bergmann *et al.*, 2004; Wang *et al.*, 2007; Meng *et al.*, 2012). Changes in the structure of receptor complexes upon ligand binding and the mechanism of signal transmission from ERfs to YODA are currently not clear.

**Fig. 1.**
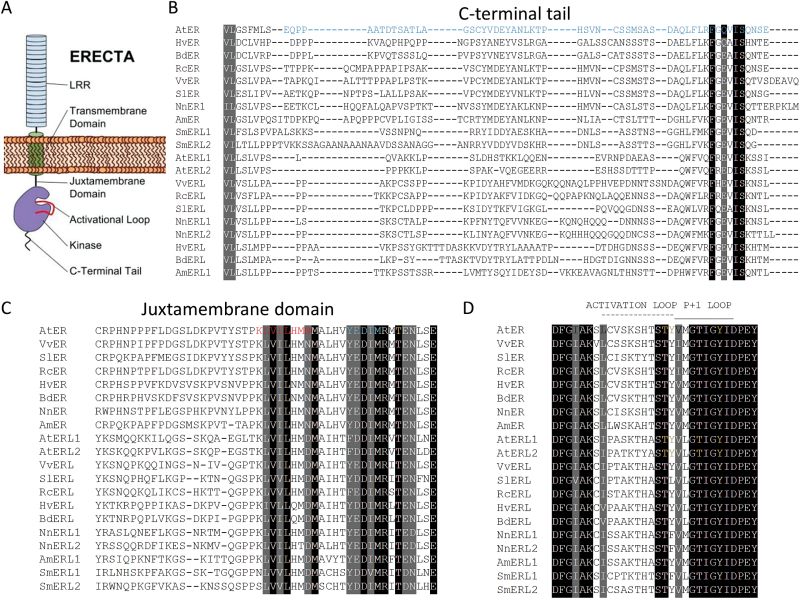
Comparison of amino acid sequences of ERECTA family proteins in different species. (A) Domain structure of the ERECTA receptor. (B–D) MAFFT alignment of the predicted amino acid sequences of *ERECTA* family genes from *Arabidopsis thaliana* (At), *Vitis vinifera* (Vv), *Solanum lycopersicum* (Sl), *Ricinus communis* (Rc), *Hordeum vulgare* (Hv), *Brachypodium distachyon* (Bd), *Nelumbo nucifera* (Nn), *Amborella trichopoda* (Am), and *Selaginella moellendorffii* (Sm). Residues that are identical among the sequences are shown with a black background, and those that are similar among the sequences are shown with a gray background. (B) The C-terminus. The blue residues have been deleted in pPZK110 and in pPZK111. (C) The juxtamembrane domain. The red residues have been deleted in pPZK104, the blue residues in pPZK105. Threonine in yellow has been substituted with Ala in pPZK102. (D) The activation loop. The predicted phosphorylation sites according to the Arabidopsis Protein Phosphorylation Site Database (PhosPht) are in yellow.

ERf protein structure consists of an extracellular leucine-rich domain (LRR), a single-span transmembrane domain, and a cytoplasmic Ser/Thr kinase domain flanked by a juxtamembrane domain (JMD) and a C-terminal tail (Fig. 1A). Previously, it was shown that the cytoplasmic segment of ERECTA is functionally important as its deletion leads to a dominant negative phenotype (Shpak *et al.*, 2003). To gain a better understanding of how ERECTA activates downstream signaling, we performed a structure–function analysis of this domain. These studies demonstrated that the cytoplasmic JMD is important for ERECTA function, but the C-terminal tail is not. Our experiments further confirmed that ERECTA is a functional kinase and suggested that Thr807, Thr812, Tyr815, and Tyr820 in the activation segment of the kinase domain are functionally important. Based on our results, we hypothesize that phosphorylation of ERECTA at Thr812 might have a stimulatory effect on receptor activity, and at Tyr815 and Tyr820 an inhibitory effect. Our experiments also indicated that the molecular mechanism of ERECTA function is different during regulation of stomata development and in elongation of above-ground organs.

## Materials and methods

### Plant material and growth conditions

The Arabidopsis ecotype Columbia (Col) was used as the wild-type (WT). The *er-105* and *er-105 erl1-2 erl2-1* mutants have been described previously (Torii *et al.*, 1996; Shpak *et al.*, 2004). Plants were grown on a soil mixture of a 1:1 ratio of Promix PGX (Premier Horticulture Inc.) and Vermiculite (Pametto Vermiculite Co.) and were supplemented with Miracle-Gro (Scotts) and approximately 3.5mg cm^–3^ of Osmocoat 15-9-12 (Scotts). All plants were grown at 20 °C under long-day conditions (18 h light/6 h dark).

### Generation of transgenic plants

In all plasmids except pPZK111 the substitutions/deletions were introduced into the genomic *ERECTA-RLUC* sequence by overlap extension PCR using pESH427 as a template (Karve *et al.*, 2011). The amplified fragments were digested with PstI, inserted into pESH427, and sequenced. The constructs carry the endogenous *ERECTA* promoter and the 35S terminator. The pPZK111 was generated by overlap extension PCR using pKUT196 as a template (Godiard *et al.*, 2003). The amplified fragment was digested with PstI, inserted into pKUT196, and sequenced. This construct carries the endogenous ERECTA promoter and terminator. The backbone of all plasmids is the vector pPZP222. The plasmids were introduced into *Agrobacterium tumefaciens* strain GV3101/pMP90 by electroporation, and into Arabidopsis *er-105* and *er-105 erl1-2/+ erl2-1* plants by vacuum infiltration. The transgenic plants were selected based on gentamicin resistance and the number of rescued lines has been quantified based on general plant morphology (Supplementary Tables S1 and S2). The *er-105 erl1-2 erl2-1* mutants were selected based on kanamycin resistance and the homozygous status of the *erl1-2* mutation was confirmed by PCR with the primers erl1g3659 (GAGCTTGGACATATAATC), erl1g4411.rc (CCGGAGAGATTGTTGAAGG), and JL202 (CATTTTATAATAACGCTGCGGACATCTAC). In addition, for transgenic lines transformed with pPZK102, pPZK110, and pPZK111 constructs, the homozygous status of the *erl1-2* mutation was confirmed by analysis of kanamycin resistance in the progeny. The quantitative phenotypic analysis of *er erl1 erl2* plants transformed with the described constructs has been done in T3 generation once their genetic status was established.

### Measurement of *Renilla* luciferase activity

ERECTA-RLUC protein expression was measured by monitoring *Renilla* luciferase activity with a 20/20n single-tube luminometer in T1 inflorescences or in T2 8-d-old seedlings using the *Renilla* Luciferase Reporter Assay (Promega). The protein concentration in each sample was determined using the Bradford assay.

### Analysis of mutant phenotypes

Measurements of stomata index and clustering were done on the abaxial side of cotyledons from 17-d-old seedlings using differential interference contrast (DIC) microscopy. For DIC, seedlings were incubated in a solution of 9:1 ethanol:acetic acid overnight, rehydrated with an ethanol series to 50% (v/v) ethanol, and then cleared in a mixture of 8:1:1 chloral hydrate:distilled water:glycerol.

### Immunoblot analysis

The crude microsomal proteins were isolated from 11-d-old WT and T2 T807D seedlings (~0.4g per sample) using a method described by Zhang *et al.* (2011). The last step of this method, an enrichment for plasma membrane proteins, was omitted. Immunoblot analysis was performed as previously described with minor modifications (Shpak *et al.*, 2003). Proteins were run on 8% or 10% SDS-PAGE. Primary anti-BAK1 polyclonal antibodies (Agrisera) were used at a dilution of 1:5000 followed by the secondary HRP Conjugated Goat Anti-Rabbit IgG antibody (Agrisera) at a dilution of 1:10 000. Primary anti-Rluc monoclonal antibodies (Millipore; clone 5B11.2) were used at a dilution of 1:5000 followed by the secondary HRP Conjugated Goat Anti-Mouse IgG antibody at a dilution of 1:7500. The detection of HRP was performed with a SuperSignal West Pico Rabbit IgG detection kit (Pierce).

### Sequence alignment

Full-length amino acid sequences of ERECTA family proteins from different species were retrieved from the NCBI database and aligned using ClustalW2 (http://www.ebi.ac.uk/Tools/msa/clustalw2/).

### Arabidopsis Genome Initiative

Arabidopsis Genome Initiative numbers for the genes discussed here are as follows: *ER* (At2g26330), *ERL1* (At5g62230), and *ERL2* (At5g07180).

## Results

### The juxtamembrane domain (JMD) is important for ERECTA function, but the C-terminal tail is not

The activity of a RLK’s kinase domain is often modulated by the flanking regions: the JMD and the C-terminal tail. In some receptors those regions inhibit kinase function, in others they are essential for the enzymatic activity (Wang *et al.*, 2005*b*; Oh *et al.*, 2009*b*, 2014). Phosphorylation of residues within these regions can often alter their function. For example, phosphorylation of Ser and Thr residues in the BRI1 C-terminal tail disables its inhibitory role (Wang *et al.*, 2005*b*).

**Fig. 2.**
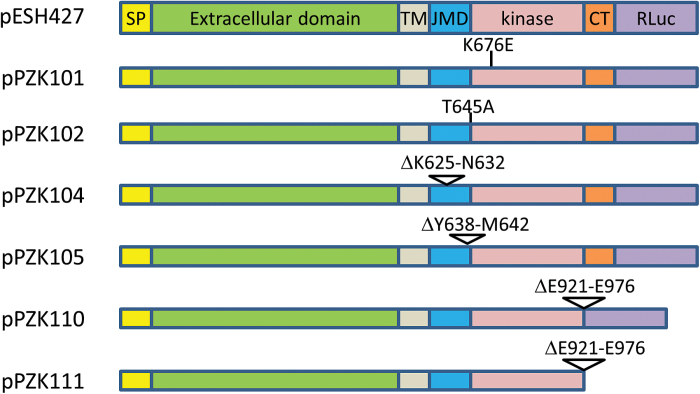
Schematic diagram of modifications introduced into the ERECTA protein. Triangles indicate deletions and lines indicate point mutations. SP, signal peptide; TM, transmembrane domain; JMD, juxtamembrane domain; CT, C-terminal tail; RLuc, *Renilla* Luciferase. In the constructs the genomic sequence of ERECTA is under the control of its native promoter and the 35S terminator. On the left are the names of the plasmids.

To examine whether regions flanking the ERECTA kinase domain have specific function, we created multiple constructs with modified genomic ERECTA sequences under the control of the native promoter (Fig. 2). With the exception of one (pPZK111), all constructs contained *Renilla* Luciferase (RLUC) at the C-terminus of the receptor to monitor the level of protein expression. The luciferase assay is a fast, reliable, and relatively cheap method to measure protein levels. Most significantly, it reflects the protein concentration in Arabidopsis extracts (Ramos *et al.*, 2001; Subramanian *et al.*, 2006). Protein titration assays and immunoblot analysis confirmed that RLUC activity reflects the level of ERECTA-RLUC accumulation in transgenic seedlings (Supplementary Fig. S1).

**Fig. 3.**
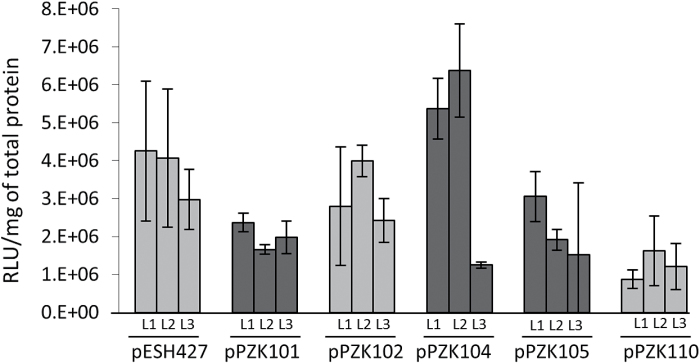
ERECTA-RLUC is expressed in the majority of transgenic lines. The level of ERECTA-RLUC expression was determined by measuring luciferase activity per milligram of total protein in inflorescences of T1 transgenic plants. RLU indicates relative light units. The mean of three biological replicates is plotted; error bars represent the SD. Three independent transgenic lines (L1–L3) were analyzed. The lines that rescue the *er-105* phenotype are in light grey and the lines that do not are in dark grey.

The unmodified ERECTA fused to RLUC (construct pESH 427) was used as a positive control. The constructs were transformed into *er-105* and into *er erl1/+ erl2* mutants and multiple independent transgenic lines were analyzed. Interestingly, we observed a decreased frequency of complementation in the T1 generation for constructs containing RLUC (Supplementary Tables S1 and S2). In our earlier experiments, the genomic ERECTA (pKUT196) rescued 100% of transgenic *er-105* plants in the T1 generation (Karve *et al.*, 2011), while this time only 27% of T1 plants were rescued by ERECTA-RLUC (pESH427). Similarly, ΔE921-E976 ERECTA (pPZK111) rescued 58% of T1 *er-105* plants while ΔE921-E976 ERECTA-RLUC (pPZK110) rescued only 16% (Supplementary Table S1). As a result, the frequency of complementation in the T1 generation has not been used as a measure of a construct functionality. Instead, for each construct we analyzed multiple T1 plants with the goal of finding three to four independent transgenic lines with relatively similar protein expression. While analysis of protein expression detected a variation in the amount of ERECTA produced in different transgenic lines, the general ability of a construct to rescue the ERECTA phenotype did not correlate with the level of protein expression in selected lines (Fig. 3). For example, expression of ERECTA in non-complemented pPZK101, pPZK104, and pPZK105 transgenic lines is equal to or higher than that in complemented pPZK110 lines. Thus, we concluded that the inability of constructs to rescue *er-105*, *er erl2*, and *er erl1/+ erl2* mutants was due to modification of ERECTA structure and not to poor expression of the protein.

**Fig. 4.**
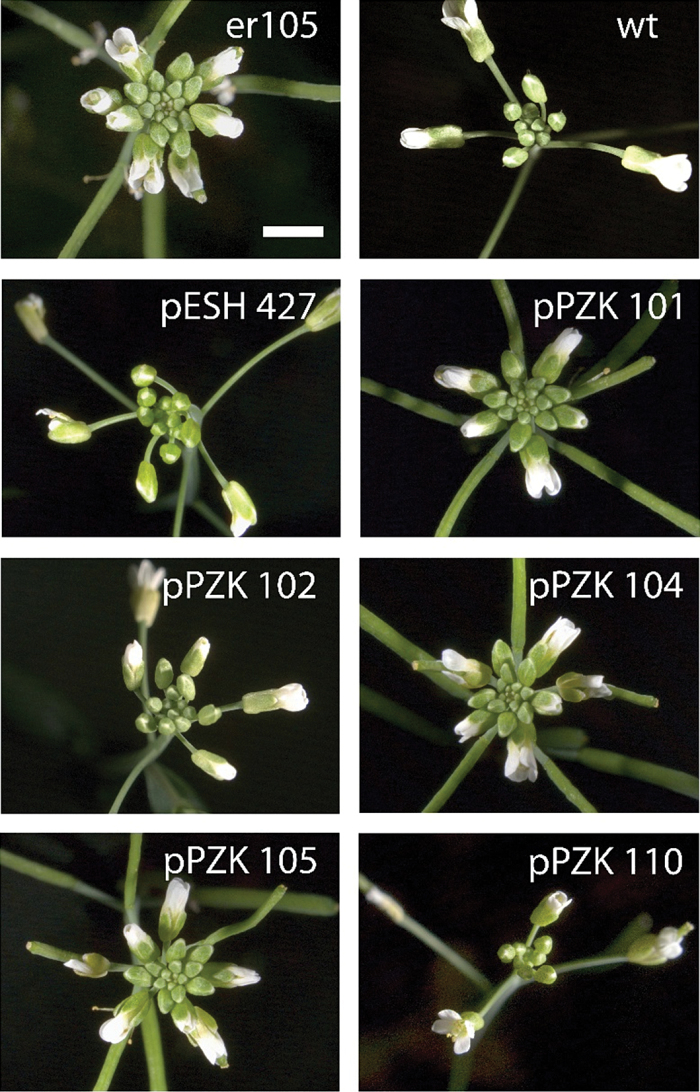
Inflorescence architecture reflects functionality of modified ERECTA receptors. Representative images of inflorescence apices of the wild-type (wt), er-105, and selected transgenic lines. All constructs were transformed into *er-105*. Scale bar =3 mm.

**Fig. 5.**
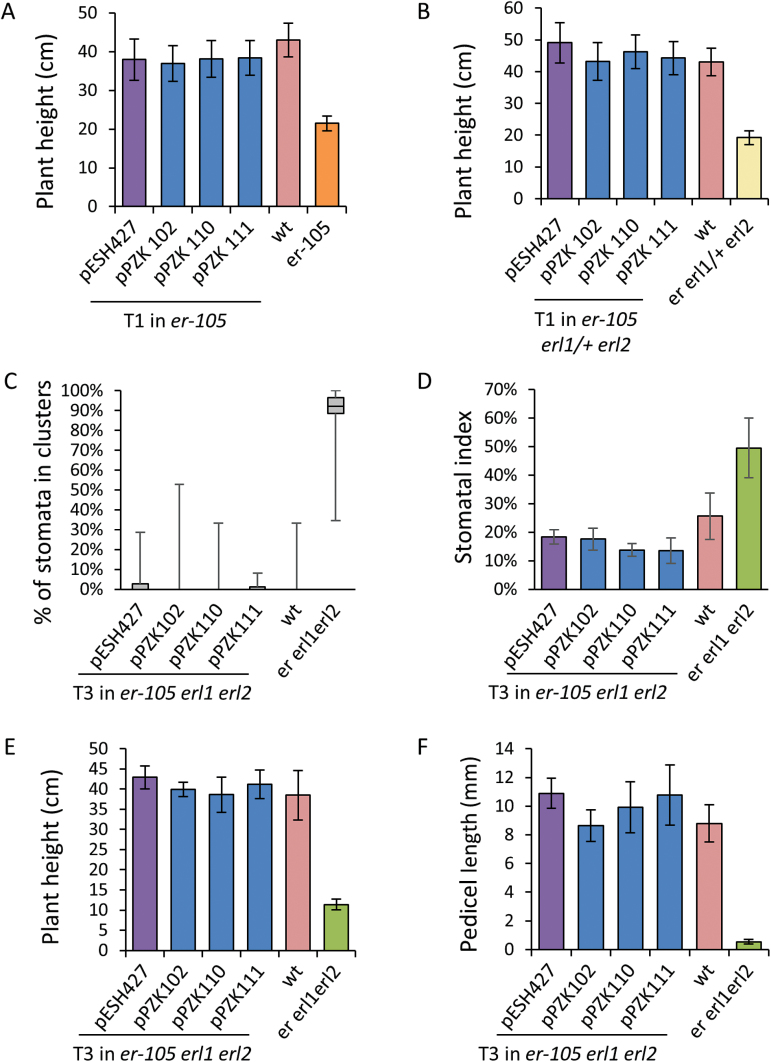
Deletion of the C-terminus domain (pPZK110 and pPZK111) or a point mutation in the JMD (T645A; pPZK 102) does not alter ERECTA’s ability to regulate stomata development or above-ground organ elongation. (A, B) Height of mature plants (A, *n*=11–34; B, *n*=8–29). (C–F) Constructs were transformed into *er erl1/+erl2* mutants and transgenic *er erl1 erl2* plants were analyzed in the T3 generation. In (C) the median is indicated as a thick horizontal line, upper and lower quartiles are represented by the top and the bottom of the boxes, and the vertical lines designate the maximum and the minimum. Epidermal phenotypes were analyzed on the abaxial side of 17-d-old cotyledons (*n*=8–13). (E.) Height of mature plants (*n*=9–18). (F) Length of mature pedicels on the main stem (*n*=80; eight measurements per stem). In (A, B, D–F) values are means ± SD.

An analysis of ERf sequences from a broad variety of angiosperms suggests low conservation of the C-terminal tail except for a short stretch of amino acid residues at the very end (Fig. 1B). Two constructs (pPZK110 and pPZK111) were created to examine the role of the C-terminal tail in ERECTA function. In both constructs the last 56 amino acids of ERECTA were deleted; in pPZK110, ERECTA was fused with RLUC and in pPZK111 it was not (Fig 2). We were concerned that RLUC at the C-terminus might interfere with receptor function and that its presence could conceal any possible increased activity of ERECTA without the C-terminus tail. However, both constructs rescued inflorescence structure and plant height of *er-105* and *er erl1/+ erl2*, similar to the positive control pESH427 (Fig. 4; Fig. 5A, B; Supplementary Table S1). In addition, pPZK110 and pPZK111 fully rescued stomata development, plant height, and pedicel length phenotypes of the *er erl1 erl2* mutants (Fig. 5C–F). And while the stomatal index in the pPZK110 and pPZK111 *er erl1 erl2* lines was reduced below wild-type levels, it was not statistically significantly different from that in the pESH427 line and therefore this decrease cannot be due to the absence of the C-terminus tail (Fig. 5D). Thus, the ERECTA C-terminus seems to be dispensable for regulation of plant architecture and stomata formation.

**Fig. 6.**
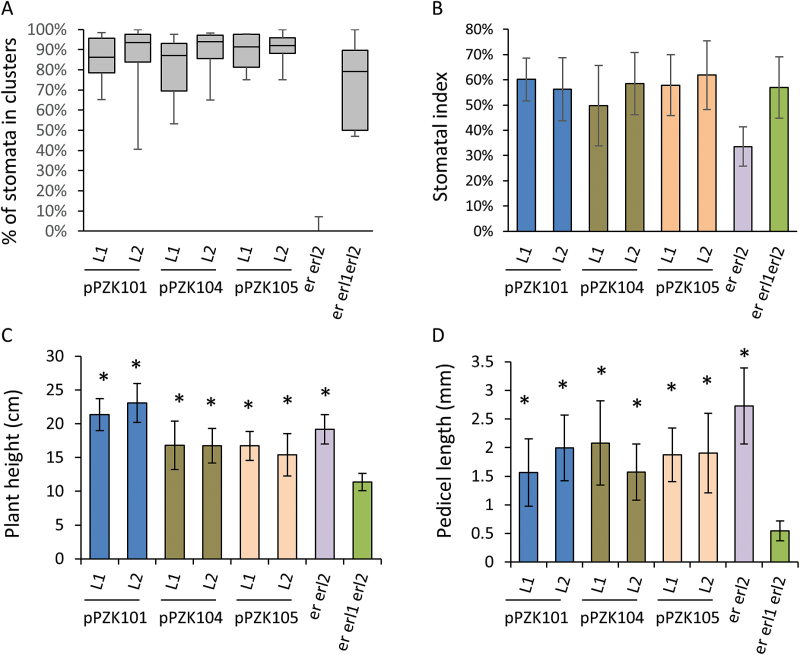
While substitution of the conserved lysine residue (K676E) in the ATP binding site of the ERECTA kinase domain (pPZK101) or deletion of short JMD segments (pPZK104 and pPZK105) disrupt ability of ERECTA to rescue stomatal phenotypes of *er erl1 erl2*, those constructs partially rescue elongation of above-ground organs. Constructs were transformed into *er erl1/+ erl2* mutants and two independent transgenic lines in the *er erl1 erl2* background were analyzed in the T3 generation. In (A) the median is indicated as a thick horizontal line, upper and lower quartiles are represented by the top and the bottom of the boxes, and the vertical lines designate the maximum and the minimum. (A, B) Epidermal phenotypes were analyzed on the abaxial side of 17-d-old cotyledons (*n*=8–16). (B–D) Values are means ± SD. (C) Height of mature plants (*n*=12–21). (D). Lengths of mature pedicels on the main stem (*n*=80; eight measurements per stem). (C, D) Values significantly different from *er erl1 erl2* (*P*<0.00001) are indicated by asterisks.

The JMD of the ERf receptors is 46–49 amino acids long. Comparison of this domain in different species revealed low conservation of the N-terminal half and high conservation of the C-terminal half (Fig. 1C). Several secondary structure prediction programs suggested the presence of a β-sheet and an α-helix in the conserved region of the JMD (Supplementary Fig. S2). Two constructs were created: one with eight residues deleted in the region of a potential β-sheet (pPZK104), and another with five residues deleted in the region of a potential α-helix (pPZK105) (Fig. 2). While ERECTA containing these modifications was expressed, it did not rescue the elongation phenotype of above-ground organs in the *er-105* mutant (Fig. 3, Fig. 4). The deletions in the JMD also abolished the ability of ERECTA to inhibit stomata formation and to regulate stomata spacing (Fig. 6A, B).

The phosphorylation and de-phosphorylation of JMD residues often regulates activity of RLKs, impinging on their enzymatic function or their ability to interact with downstream targets (Aifa *et al.*, 2006; Heiss *et al.*, 2006; Thiel and Carpenter, 2007; Chen *et al.*, 2010). In Erf, the JMD contains only one conserved Thr (Thr645) and no conserved Ser or Tyr residues (Fig. 1C). This Thr is conserved not only in ERf but also in many other PELLE/RLK kinases (Supplementary Fig. S3). In Pto and XA21, this Thr plays an important biological function and is essential for their autophosphorylation (Sessa *et al.*, 2000*a*; Chen *et al.* 2010). The *Arabidopsis thaliana* phosphorylation site database PhosPhAt predicts phosphorylation of Thr645 (Durek *et al.*, 2010). To test whether Thr645 is important for ERECTA function, this residue was substituted with Ala in the construct pPZK102 (Fig. 2). This substitution did not alter ERECTA functionality and the construct rescued organ elongation defects in *er-105* (Fig. 4, Fig. 5A, Supplementary Table S1), *er-105 erl1/+ erl2* (Fig. 5B), and *er-105 erl1 erl2* (Fig. 5E, F, Supplementary Table S1). In addition, the pPZK102 construct fully rescued stomata formation defects in the *er erl1erl2* mutant (Fig. 5C, D). These data suggest that Thr645 is not essential for ERECTA function. While our data suggest that the JMD is essential for ERECTA functionality, we were unable to identify critical phosphorylation sites in this region.

### Importance of the kinase domain for ERECTA function

*In vitro* ERECTA is a weak kinase (Lease *et al.*, 2001, Meng *et al.*, 2015). The phenotypes of several mutants with substitutions and deletions in the ERECTA kinase domain suggest that the ability to phosphorylate might be important for ERECTA function (Lease *et al.*, 2001). Alternatively, these mutations might lead to receptor instability or change ERECTA’s capacity to bind co-receptors or downstream targets. The mutations are in the different α-helixes of the kinase domain and the exact function of those amino acids is not known. To further test whether the ability to phosphorylate is important for ERECTA function, a conserved lysine in the ATP-binding domain was replaced with a glutamate (K676E; pPZK101; Fig. 2). We identified several pPZK101 transgenic lines in the *er-105* background with sufficient expression of ERECTA (Fig. 3). However, organ elongation defects were not rescued in those lines (Fig. 4). The pPZK101 construct was also unable to rescue epidermal phenotypes in the *er-105 erl1 erl2* mutant (Fig. 5C, D). Thus, ERECTA is likely an active kinase *in vivo* and its ability to phosphorylate has functional significance.

The PhosPhAt database predicts multiple phosphorylation sites in the kinase domains of ERECTA, ERL1, and ERL2. Based on these predictions and evolutionary conservation, two residues were selected for alanine substitutions preventing phosphorylation: T823, a residue at the end of λEF helix, and T906, a residue in the αI helix. T823 of ERECTA is homologous to T872 of the receptor-like kinase HAESA, a residue phosphorylated *in vitro* and contributing to enzymatic activity of HAESA in *vitro* (Taylor *et al.*, 2016). However, these substitutions did not disrupt functionality of ERECTA (Supplementary Fig. S4).

**Fig. 7.**
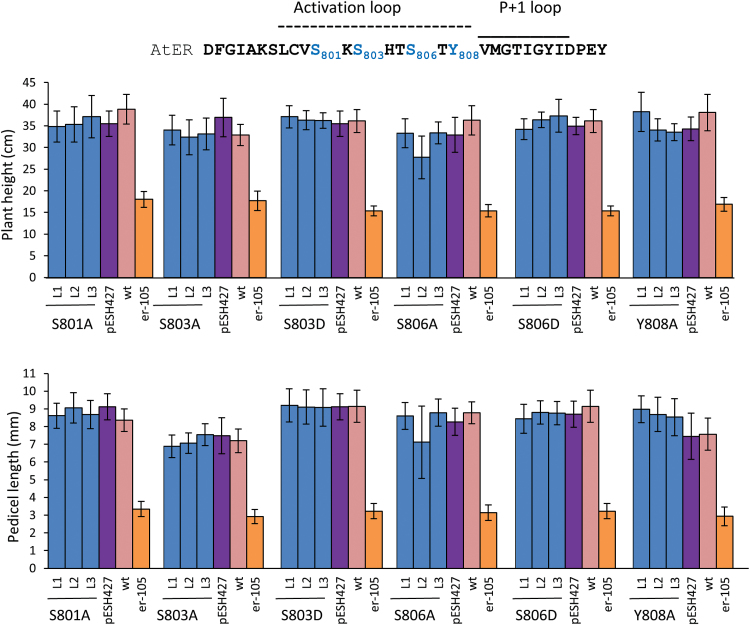
Site-directed mutagenesis of four potential phosphorylation sites in the activation loop of ERECTA suggests that these residues are not critical for ERECTA function. To determine ERECTA functionality, the constructs were transformed into *er-105*, and the height of mature plants (*n*=9–18) and the length of pedicels on the main stem (*n*=40; eight measurements per stem) were measured. Error bars represent ±SD. Three independent transgenic lines (L1–L3) were analyzed in the T2 generation. The mutated residues are in blue in the sequence at the top.

**Fig. 8.**
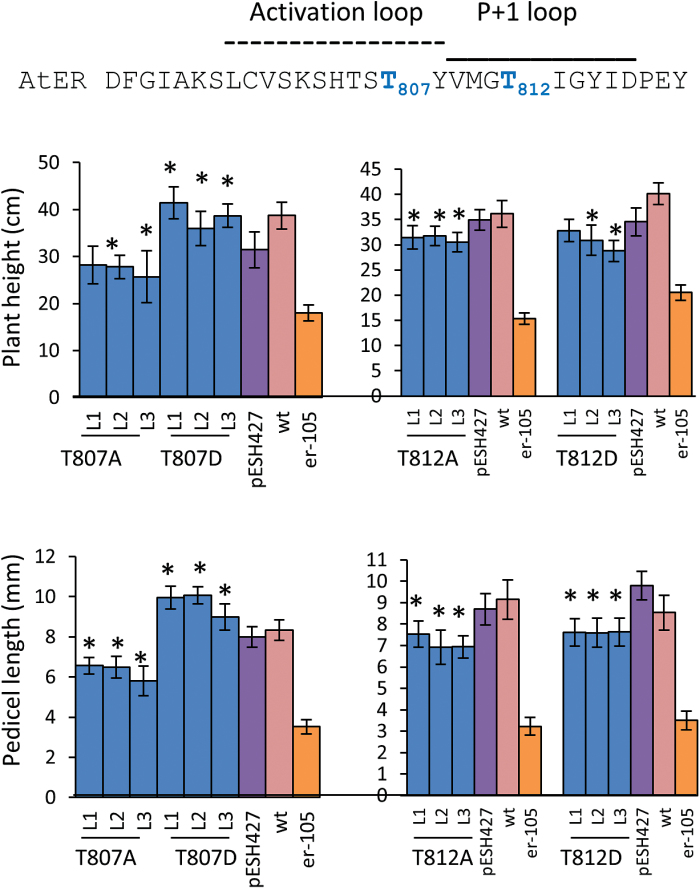
Site-directed mutagenesis of two conserved threonines in the activation segment impairs ERECTA function. To determine ERECTA functionality, the constructs were transformed into *er-105*, and the height of mature plants (*n*=9–18) and the length of pedicels on the main stem (*n*=40; eight measurements per stem) were measured. Error bars represent ±SD. Three independent transgenic lines (L1–L3) were analyzed in the T2 generation. The mutated residues are in blue in the sequence at the top. The transgenic line values significantly different from pESH427 (*P*<0.005) are indicated by asterisks.

Next, we analyzed multiple Ser/Thr/Tyr in the activation segment by substituting them to Ala or as a phosphomimic to Asp. Alanine and aspartate substitution of Ser801, Ser803, Ser806, and Tyr808 in the activation loop did not have any effect on ERECTA function in control of organ elongation (Fig. 7). Substitutions of Thr812 to Ala and to Asp slightly, but statistically significantly, reduced functionality of ERECTA (Fig. 8). ERECTA with these substitutions was not able to fully rescue elongation defects of pedicels and stems when transformed into the *er-105* mutant. These substitutions did not alter the expression level of ERECTA (Supplementary Fig. S5). Interestingly, these two substitutions had a very similar negative impact on ERECTA function, and the phenotype of plants expressing T812A and T812D did not differ statistically. Therefore, phosphorylation of Thr812 is unlikely to play a major role in the activation of ERECTA.

Another residue that is important for ERECTA function is Thr807. The T807A substitution substantially reduced functionality of ERECTA, while organ elongation in plants expressing ERECTA with T807D substitution was similar to the wild-type or even greater (Fig. 8). Based on these data, we speculate that phosphorylation of Thr807 might have a positive impact on ERECTA function.

**Fig. 9.**
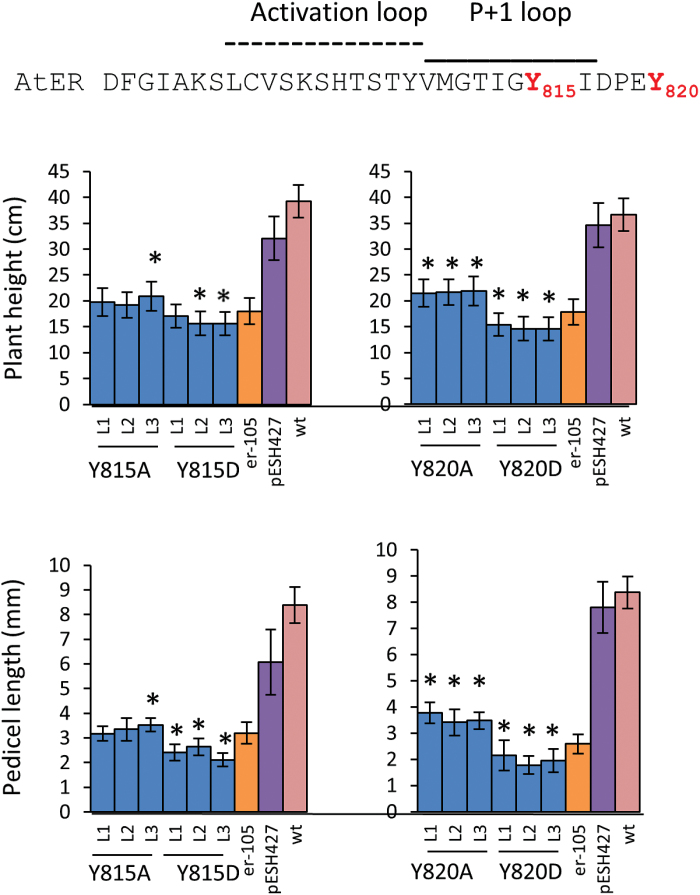
Site-directed mutagenesis of two conserved tyrosines in the activation segment of ERECTA suggests a negative role of their potential phosphorylation. To determine ERECTA functionality, the constructs were transformed into *er-105*, and the height of mature plants (*n*=9–18) and the length of pedicels on the main stem (*n*=40; eight measurements per stem) were measured. Error bars represent ±SD. Three independent transgenic lines (L1–L3) were analyzed in the T2 generation. The mutated residues are in red in the sequence at the top. The transgenic line values significantly different from *er-105* (*P*<0.005) are indicated by asterisks.

Finally, we observed that two Tyr substitutions had a very strong impact on plant growth. Both Y815A and Y820A strongly reduced ERECTA functionality, but were statistically different from *er-105*, suggesting that with those substitutions ERECTA retained a very low level of functionality (Fig. 9). Interestingly, substitutions of these Tyr to Asp resulted in a dominant negative phenotype (Fig. 9). Plants expressing ERECTA with Y815D or with Y820D were statistically shorter compared to *er-105*. This result suggests that these two Tyr are critical for ERECTA functionality and their phosphorylation might have a negative impact on ERECTA function.

### Distinct signaling mechanisms of ERECTA in multiple developmental pathways

**Fig. 10.**
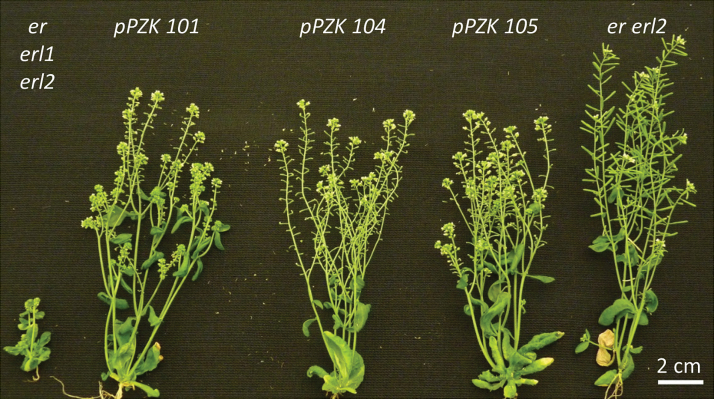
pPZK101, pPZK104, and pPZK105 constructs can partially rescue elongation of above-ground organs in the *er erl1 erl2* mutant. Representative 6-week-old plants from left to right: *er erl1 erl2*; T3 *pPZK101*, *pPZK104*, and *pPZK105* in *er erl1erl2* background; *er erl2*.

While deletions in the JMD (pPZK 104 and pPZK 105) or disruption of the kinase activity by the K676E substitution (pPZK 101) destroyed ERECTA’s ability to regulate stomata development and fertility in *er erl1 erl2* or to rescue *er*, *er erl2*, and *er erl1/+ erl2* morphogenetic defects, these constructs were able to partially rescue stem and pedicel elongation in the *er erl1 erl2* mutant (Fig. 6C, D; Fig. 10). In Fig. 6C, D the plant height and pedicel length are compared in fully mature plants. Because *er erl1 erl2* grows slower and for a longer period of time, the plants are of different ages. If we compare plants of similar age as in Fig. 10, the ability of pPZK101, pPZK 104, and pPZK105 to partially rescue the *er erl1 erl2* mutant becomes even more obvious. We speculate that in the *er* and *er erl2* backgrounds the ability of pPZK101, pPZK 104, and pPZK 105 to alter organ elongation is not evident due to much stronger impact of ERL1 and ERL2 on plant growth. Taken together, these results suggest that the signal transduction by ERECTA is different in organ elongation versus control of stomata formation and development of flower organs.

## Discussion

Considerable progress has been made recently in understanding the composition of receptor–ligand complexes formed by ERf in the plasma membrane (Lee *et al.* 2012, 2015). However, the mechanism of the cytoplasmic domain activation and transmission of the signal by ERf is still unclear. To explore the significance of ERECTA’s kinase domain and the mechanism of its activation, we used a structure–function approach.

**Fig. 11.**
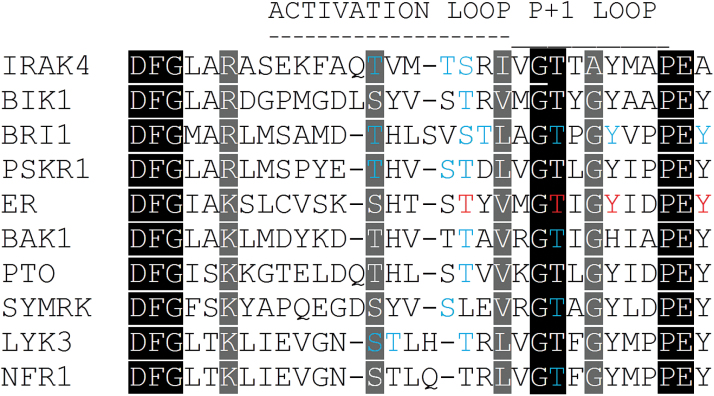
Alignment of the activation segments from plant receptor-like kinases ER, BAK1, LYK3, NFR1, BRI1, and PSKR1, plant kinases BIK1 and PTO, and human kinase IRAK4. Functionally significant amino acids are in red. Amino acids that are phosphorylated (either *in vitro* or *in vivo*) and functionally significant *in planta* or essential for the enzymatic activity are in blue. Residues that are identical among the sequences are given a black background, and those that are similar among the sequences are given a gray background.

ERECTA is a RD kinase, which means that it has a conserved arginine (R) immediately preceding an aspartate (D) in the catalytic loop. Previous research has established that ERECTA is a weak kinase *in vitro* (Lease *et al.*, 2001; Meng *et al.*, 2015). Accordingly, we observed that ERECTA with substitution of the conserved lysine in the ATP-binding domain is unable to rescue the majority of developmental defects in mutants, and therefore the ability to phosphorylate is essential for ERECTA function. While inactive kinases adopt a variety of distinct conformations, their activation often depends on a change in the structure of the activation segment, which in the RD kinases is the primary site of regulatory phosphorylation (Johnson *et al.*, 1996). The activation segment is variable in length and sequence but it is restricted by highly conserved DGF and APE motifs, and in Ser/Thr kinases it almost always contains a characteristic GlyThr or GlySer dipeptide motif (Fig. 11). In Arabidopsis, more than 99% of RD LRR RLKs have a Thr or Ser in this motif (Wang *et al.*, 2005*a*). In the active state, the hydroxyl group of the threonine or serine from the GlyThr/Ser motif forms hydrogen bonds with the catalytic aspartate of the HRD motif and the lysine one nucleotide behind this motif (Nolen *et al.*, 2004). In IRAK4 and many other mammalian Ser/Thr kinases this Thr is important for the kinase activity, but is not a major phosphorylation site (Wang *et al.*, 2009; Bayliss *et al.*, 2012). Alanine or phosphomimetic substitutions of homologous Thr/Ser in plant kinases such as SERK1 (T468A, T468E), BRI1 (T1049A), SYMRK (T760A), BAK1 (T455A, T455D, T455E), BIK1 (T242A), ACR4 (T681A, T681D), HAESA (S861A), and PSKR1 (T899A) lead to loss of kinase function (Shah *et al.*, 2001; Wang *et al.*, 2005*a*, 2008; Yoshida and Parniske, 2005; Laluk *et al.*, 2011; Meyer *et al.*, 2011; Taylor *et al.*, 2013; Hartmann *et al.*, 2015). In BRI1, FLS2 (T1040A), BAK1, BIK1, and HAESA these substitutions were shown to decrease functionality of receptors *in planta* (Wang *et al.*, 2005*a*, 2008; Robatzek *et al.*, 2006; Laluk *et al.*, 2011; Taylor *et al.*, 2016). While our work demonstrates that both alanine or phosphomimetic substitutions of Thr812 alter functionality of ERECTA, the effect is surprisingly small. In this respect ERECTA resembles the RD receptor-like kinases PSKR1 and FERONIA where substitution of homologous S701 and T899, respectively, does not disrupt receptor function (Hartmann *et al.*, 2015; Kessler *et al.*, 2015).

Thr/Ser residues preceding the GlyThr motif are the primary phosphorylation sites that are essential for the activation of mammalian Ser/Thr kinases (Nolen *et al.*, 2004; Bayliss *et al.*, 2012). For example, the kinase activity of IRAK4 is regulated by the autophosphorylation of three sites, Thr342, Thr345, and Ser346, located in front of the GlyThr motif (Cheng *et al.*, 2007). Based on structural studies, phosphorylation of Thr345 is responsible for the activation of IRAK4 kinase while phosphorylation of Thr342 and Ser346 might stabilize the activation loop in the active state (Kuglstatter *et al.*, 2007). Current literature suggests that residues homologous to Thr345 or Ser346 in IRAK4 are likely to be the primary phosphorylation sites in plant Ser/Thr kinases. Thus, Thr237 of BIK1 is necessary for full kinase activity and is a major phosphorylation site in response to flg22 (Lu *et al.*, 2010; Laluk *et al.*, 2011). In LYK3, T475A substitution leads to decreased kinase activity (Klaus-Heisen *et al.*, 2011). The equivalent Thr233 in Pti1 is the major site of autophosphorylation and phosphorylation by Pto kinase (Sessa *et al.*, 2000*b*). In BAK1, Thr450 is phosphorylated and T450A substitution reduces functionality of the receptor (Wang *et al.*, 2008). Crystal structure confirmed the significance of T450 phosphorylation for enzymatic activity of BAK1 (Yan *et al.*, 2012). Ser1044 is phosphorylated in BRI1 and, out of all Ser/Thr to Ala substitutions in the activation segment, the S1044A substitution has the most severe negative impact on BRI1 functionality (Wang *et al.*, 2005*a*, 2016). S856 in the activation segment of HAESA is a phosphorylation site that positively regulates kinase activity and contributes to the functionality of the receptor (Taylor *et al.*, 2016). Thr807 of ERECTA is homologous to Ser346 of IRAK4 (Fig. 11). Therefore, it is not surprising that out of all Ser/Thr substitutions in the activation segment of ERECTA only T807A substitution significantly reduces receptor functionality, and ERECTA with a T807D substitution is fully functional. Based upon this data, we speculate that Thr807 is the primary phosphorylation site in the activation segment of ERECTA. Future structural and biochemical studies will be essential to confirm this hypothesis. In general, our findings resemble those obtained for HAESA where substitutions of only two Ser/The residues in the activation segment disrupt receptor function (Taylor *et al.*, 2016). Those residues are homologous to Thr807 and Thr812 of ERECTA, although in ERECTA the substitution of T812 has a weaker effect on the receptor function.

Plant receptor-like kinases have dual specificity, phosphorylating both Ser/Thr and Tyr residues. Two tyrosine residues in the BRI1 P+1 loop, Tyr1052 and Tyr1057, have been shown *in vivo* to play an important role in BR signaling and their phosphorylation is predicted to have a negative impact on the kinase activity (Oh *et al.*, 2009*a*, 2009*b*). Residues homologous to Tyr1057 of BRI1 have been shown to be critical for the function of other receptor-like kinases. Thus, Tyr463 of BAK1 is essential to its catalytic activity (Oh *et al.*, 2010). Tyr250 in the activation segment of BIK1 can be autophosphorylated or transphosphorylated by BAK1 and is important for BIK1 function in plant defenses (Lin *et al.*, 2014). Consistent with this, we observed that substitutions of homologous Tyr residues in the P+1 loop of ERECTA, Tyr815 and Tyr820, drastically reduced functionality of the receptor. As substitutions to Ala were less severe than substitutions to Asp, we hypothesize that phosphorylation of those residues could lead to inhibition of ERECTA function.

Phosphorylation events in the JMD and the C-terminal regions often alter activity of receptor-like kinases (Wang *et al.*, 2005*b*; Oh *et al.* 2009*b*, 2014). However, this might not be the case for ERECTA: no changes in ERECTA functionality were observed after the deletion of the C-terminus. Thr645 is the only conserved Thr/Ser/Tyr in the JMD, yet its substitution had no effect on ERECTA function. The receptor-like kinase BAK1 associates with multiple receptor-like kinases including ERECTA family receptors (Meng *et al.*, 2015). When BAK1 associates with BRI1 it increases its activity by phosphorylating the JMD and the C-terminus (Wang *et al.*, 2008). The role of BAK1 during interaction with ERECTA is likely to be different as phosphorylation of ERECTA kinase’s flanking regions is not likely to be significant for its function.

A majority of the RLK/Pelle kinases have an N-terminal extension in front of the N-terminal lobe of the kinase domain (Lei *et al.*, 2005; Wang *et al.*, 2006). The N-terminal extension is often an integral part of the overall fold of kinase and is essential for its activity. For example, the N-terminal extension is required for BRI1 enzymatic activity (Oh *et al.*, 2012). While there is no sequence similarity between the N-terminal extensions of various kinases, there is some similarity of structure. The crystal structure of IRAK4 revealed a short β strand and an α-helix in the N-terminal extension region while those of BRI1 and BAK1 suggested the existence of an α-helix (Wang *et al.*, 2006; Yan *et al.*, 2012; Bojar *et al.*, 2014). Homology modeling of LYK3 predicted an α helix in the N-terminal extension region (Klaus-Heisen *et al.*, 2011). Four different programs (JPRED 4, NETSURFP, PSIPRED, and I-TASSER) predicted the existence of a short β strand and an α-helix in the N-terminal extension region of ERECTA. While we were unable to identify functionally significant putative phosphorylation sites in the JMD, our work determined that this domain is significant for ERECTA function. Two deletions in that region led to a functionally inactive receptor. This may be due to disruption of the N-terminal extension structure and, as a result, inactivation of ERECTA’s enzymatic function.

Our structure–function analysis indicates that ERECTA function differs in the specific developmental processes in which it participates. The kinase function is absolutely essential for ERECTA’s ability to regulate stomata formation and flower structure. Simultaneously, the kinase-dead ERECTA is able to partially rescue stem and pedicel elongation defects in the *er erl1 erl2* background. These results suggest that there are distinct signaling requirements for ERECTA in different developmental processes and imply that ERECTA might transmit the signal to downstream targets in different ways. The receptor-like kinases BAK1 and SCRAMBLED have also been shown to control multiple pathways using distinct signaling mechanisms with different requirements for their kinase domain function (Oh *et al.*, 2010; Kwak *et al.*, 2014). In addition, we observed that kinase-dead ERECTA and ERECTA without the cytoplasmic domain (Δkinase) function very differently. The Δkinase ERECTA confers dominant negative effects, probably titrating positive regulators of the signaling pathway through the extracellular domain (Shpak *et al.*, 2003). The kinase-dead ERECTA is partially functional in regulation of organ elongation, which hypothetically could occur through titration of negative regulators by the kinase domain.

## Supplementary Material

Supplementary Data
