# Supplementary material for: Identification of critical functional residues of receptor-like kinase ERECTA
Source: J Exp Bot. 2017 Feb 15;68(7):1507–18. doi: 10.1093/jxb/erx022 (PMC5441908; doi:10.1093/jxb/erx022)
Supplement: Supplementary Data [file erx022_Supplementary_Data.zip › supplementary_figures_S1_S5_tables_S1_S2.pdf]

A

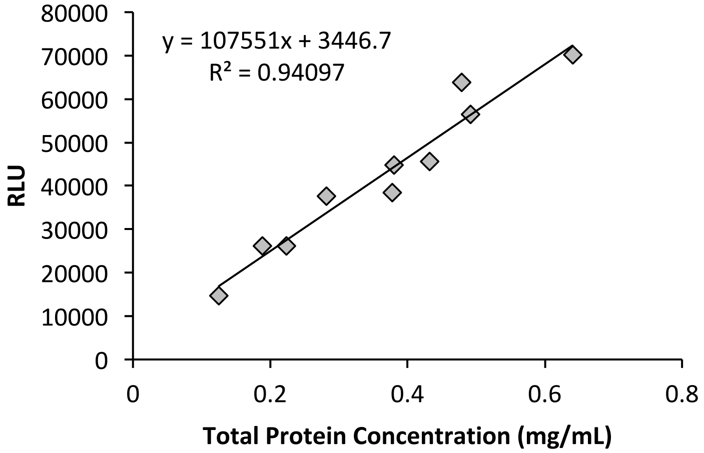

B

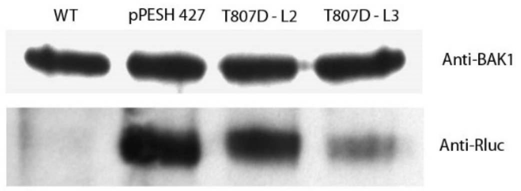

Figure S1. Both the luciferase titration curve and the immunoblot analysis confirm that the Renilla luciferase assay is a reliable method to test accumulation of RLUC-fused proteins in extracts from Arabidopsis seedlings. A. The luminescence signal increases linearly with increasing concentration of total protein in extracts from Arabidopsis seedlings expressing ERECTA-RLUC (pESH427). B. Immunoblot analysis of protein extracts from wild type seedlings and seedlings expressing ERECTA-RLUC (pESH427) and two independent lines expressing ERECTA-RLUC with T807D substitution. Accumulation of ERECTA-RLUC is consistent with the results obtained by the RLUC assay (Fig S5). The immunoblot probed with anti-BAK1 antibody reflects uniform loading of microsomal proteins.

```
CRPHNPPPFLDGSLDKPVTYSTPKLVILHMNMALHVYEDIMRMTENLSE    JMD sequence

------------------------EEEEE----HHHHHHHHHHHH----    JPRED
----------------------HHHHHHHHHHHHHHHHHHHHHHHH---    SCRATCH
------------------------EEEEEE--HHHHHHHHHHHHHH---    NETSURFP
-----------------------EEEEEE---HHHHHHHHHHHHH----    PSIPRED
-----------------------EEEEEE---HHHHHHHHHHHHHH---    I-TASSER
```

Figure S2 The secondary structure of the 49 a.a. long ERECTA JMD as predicted by five different programs: JPRED 4, SCRATCH, NETSURFP, PSIPRED and I-TASSER. H represents α-Helix, E represents extended strand and a hyphen indicates no prediction.

```
                    *
HsIRAK3        FQNIIEGTRNFHKDFLIGEGEIFEVY
LjSYMRK        LEYIEVAT-ERRYKTLIGEGGFGSVY
AtER           YEDIMRMTENLSEKYIIGHGASSTVY
HsIRAK1        LCEISRGTHNFSEELKIGEGGFGCVY
AtTMK          IQVLRSVTNNFSSDNILGSGGFGVVY
AtSTRUBBELIG   IASLQQYTNNFSEENIIGEGSIGNVY
AtFLS2         PKELEQATDSFNSANIIGSSSLSTVY
AtCERK1        LEELAKATDNFNLSFKIGQGGFGAVY
BnSRK          LETVVKATENFSNCNKLGQGGFGIVY
AtBAK1         LRELQVASDNFSNKNILGRGGFGKVY
AtEMS1         LGDIVEATDHFSKKNIIGDGGFGTVY
LpPTO          LVDLEEATNNFDHKFLIGHGVFGKVY
DmPELLE        YAELENATDGWSPDNRLGQGGFGDVY
OsXA21         YSQLVKATDGFAPTNLLGSGSFGSVY
AtBRI1         FADLLQATNGFHNDSLIGSGGFGDVY
AtPSKR         YDDLLDSTNSFDQANIIGCGGFGMVY
AtCLV1         DFKSEDVLECLKEENIIGKGGAGIVY
AtHAESA        HFSEHEIADCLDEKNVIGFGSSGKVY
AtPXY          FTADDVVECLSKTDNILGMGSTGTVY
```

Figure S3 Comparison of amino acid sequences at the N-terminus of the kinase domain in the proteins belonging to the PELLE/RLK family of kinases. Residues that are identical among the sequences are given a black background, and those that are similar among the sequences are given a gray background. The Thr residues analogous to Thr645 of ERECTA are labeled with an asterisk.

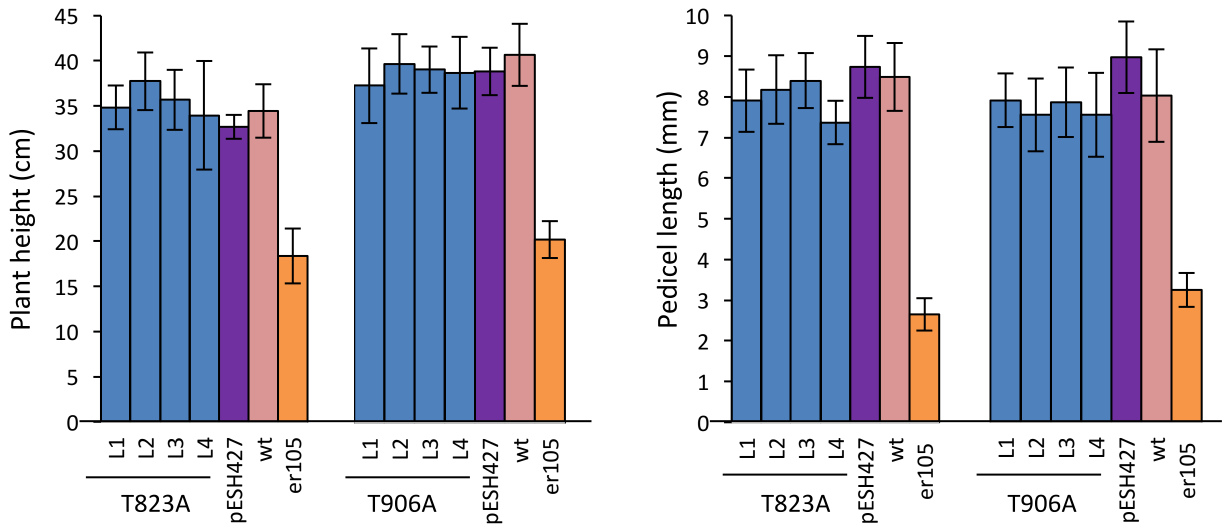

Figure S4 Site directed mutagenesis of two potential phosphorylation sites in the kinase domain of ERECTA suggests that these residues are not critical for ERECTA function. Two amino acids are predicted to be phosphorylated according to the Arabidopsis Protein Phosphorylation Site Database (PhosPht) and are conserved in ERECTA, ERL1, and ERL2. To determine ERECTA functionality the constructs were transformed into *er-105,* and the height of mature plants (n=9-18) and the length of pedicels on the main stem (n=40; eight measurements per stem) were measured. Error bars represent one SD. Four independent transgenic lines (L1-L4) were analyzed in the T2 generation.

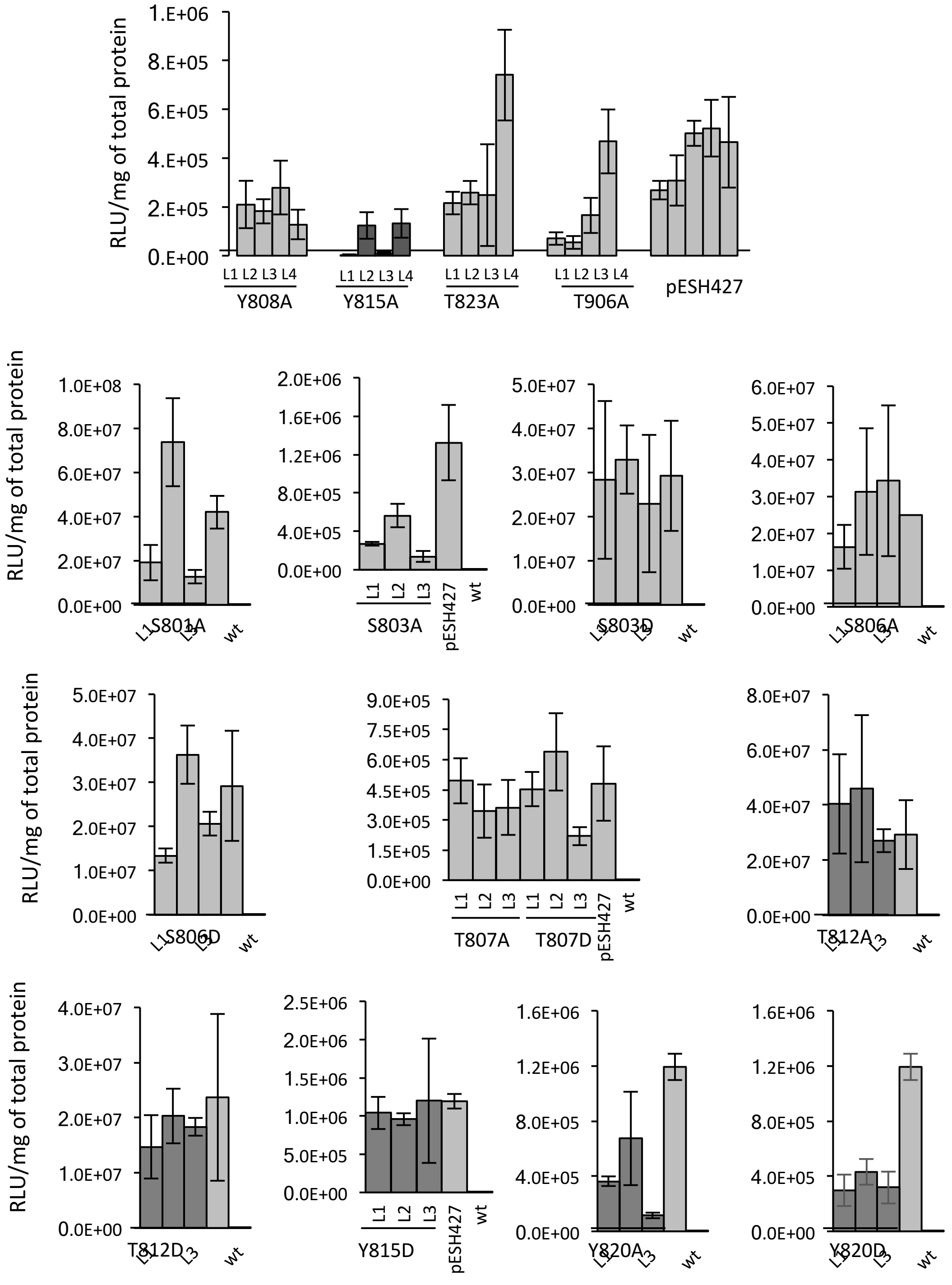

Figure S5. ERECTA-RLUC is expressed in the majority of transgenic lines. The level of ERECTA-RLUC expression was determined by measuring luciferase activity per milligram of total protein in 8 day old T2 seedlings. RLU indicates relative light units. The mean of three biological replicates is plotted; error bars represent one SD. Three to four independent transgenic lines (L1-L4) were analyzed. In light grey are lines that rescue the *er-105* phenotype and in dark grey are lines that do not. WT indicates background RLU/mg of total protein.

Name of the construct	Mutation	Transformation of *er* plants	Transformation of *er erl1/+ erl2* plants
		# of fully or partially complemented plants/ total T1 plants analyzed	# of fully or partially complemented plants/ total T1 plants analyzed
pESH 427	none	13/48	30/44
pPZK 101	K676E	0/38	0/74
pPZK 102	T645A	13/50	25/45
pPZK 104	ΔK625-N632	0/27	0/22
pPZK 105	ΔY638-M642	0/27	0/24
pPZK 110	ΔE921-E976	7/43	24/33
pPZK 111	ΔE921-E976	36/62	20/24

Table S1: Ability of constructs to partially or fully rescue mutant phenotypes. The constructs were introduced into *er-105* or *er-105 erl1-2/+ erl2-1* genotypes. When plants were transformed with pPZK102, pPZK110, or pPZK111 in T1 generation we recovered plants with the all expected genotypes, *er erl2*, *er erl1/+ erl2*, and *er erl1 erl2.* However when *er erl1/+ erl2* plants were transformed with pPZK101, pPZK104, and pPZK105 very few *er erl1 erl*2 plants were recovered in T1 due to their small size. The numbers in the table reflect ability of pPZK101, pPZK104, and pPZK105 to complement *er* (the third column) or *er erl2* and *er erl1/+ erl2* (the forth column) genotypes. The numbers in the table do not reflect ability of the constructs to rescue the *er erl1 erl2* mutant. Complementation was determined by visual observation of plant height and pedicel length.

Name of the construct	Mutation	# of complemented plants/ total T1 plants
pESH601	S801A	11/36
pESH602	S803A	5/36
pESH603	S803D	10/18
pESH604	S806A	6/24
pESH605	S806D	6/27
pPZK121	T807A	4/22
pPZK606	T807D	7/23
pESH607	T812A	10/54
pESH608	T812D	13/85
pPZK122	Y808A	8/17
pPZK123	Y815A	0/20
pESH609	Y815D	0/27
pESH610	Y820A	0/35
pESH611	Y820D	0/27
pPZK124	T823A	6/22
pPZK125	T906A	11/21

Table S2: Ability of constructs to partially or fully rescue *er* mutant phenotypes. Complementation of *er* is based on visual observation of plant height and pedicel length.
